# Some General Rules Which Will Determine the Necessity of an Operation in the Nasal Passages

**Published:** 1887-05

**Authors:** Frank H. Potter

**Affiliations:** Surgeon to the Nose and Throat Department, Good Samaritan Eye and Ear Infirmary; Assistant in *Materia Medica*, Niagara University


### *SOME GENERAL RULES WHICH WILL DETERMINE THE NECESSITY OF AN OPERATION IN THE NASAL PASSAGES.**

By FRANK H. POTTER, M. D.

Surgeon to the Nose and Throat Department, Good Samaritan Eye and Ear Infirmary;
Assistant in *Materia Medica*, Niagara University.

It is the purpose of this paper to present for your consideration some general principles which should govern the determination of an operation within the nasal passages. Of course, there is an indeterminate factor here, as in all departments of surgery, which may prevent the successful issue of any opera

*Read at the annual meeting of the Alumni Association, Medical Department, Niagara University, April, 1887.

tion. The hereditary influences and individual peculiarities always give to the personal equation of a patient serious weight; and our obscure knowledge concerning these mysterious forces should make the surgeon cautious concerning his prognosis. It is not yet possible, except within well-defined limits, to reduce to general principles rules governing these idiosyncrasies, as we call them—a term which, in my opinion, covers over a great amount of ignorance on our part concerning nature's laws. This reference to the personal equation of patients is made because I desire it to be borne in mind during the brief discussion that follows. Nothing I shall say should operate entirely against a particular case when carefully considered; but the rules propounded will, I think, assist us in determining, in the majority of instances, when surgical relief should be advised.

We can reduce the discussion of this question almost to a single proposition: When there are symptoms, operate, no matter how small may be the offending condition; when there are no symptoms, don't operate, no matter how great may be the departure, real or apparent, from the normal condition. I do not believe it should be our effort to construct a perfect anatomical and physiological nose, and then attempt to mould and re-mould all other noses until they reach, or come fairly near, that standard. Apart from the great difficulty of doing this, it is not the proper theory of practice. It is wiser to be content with removing the offending conditions, whatever they may be, trusting to the resources of nature to effect the further relief. Perhaps, in order not to be misunderstood, I should discuss this matter a little more in detail.

A patient comes to you complaining of some nasal trouble, and upon investigation you find the symptoms due to an irritation somewhere in the cavities of the nose. This irritation should be carefully located and removed. If you have reason to suspect several causes for the irritation, seek out the most offending one, remove it and wait. It is possible that nature may be able to take care of the other causes after the most serious one has disappeared. This, then, is a primary rule in the treatment of the diseases of the nose, viz.: All causes of irritation must be removed, the most offending one first if possible. In this connection, I desire to call attention to a paper read by Dr. A. B. Farnham, of Milwaukee, before the Wisconsin State Medical Society in 1886. In this paper the author arrives at practically the same conclusions as we have here expressed. Discussing the principle just mentioned, he says:

"The primary application of this principle takes out of the discussion the larger growths and limits it to the smaller hypertrophies of the mucous membranes, and to the exostoses and deviations of the system. These latter play, by all means, the leading *rôle* in the mucous hypertrophies and degenerations under consideration. From congenital and accidental causes, the growths upon, and deviations of this partition are of exceeding variety and complexity. No matter how numerous or how varied, let them alone unless they are causing irritation, and even then take away only sufficient to fully relieve the pressure and chafing of the opposing membrane. By one or more observations, determine the most offending growth, or portion of a growth, and remove that and wait, when possible, and see the full result of what has been done."

So many writers have arrived, independently, at these same conclusions, that it would only be wearisome to repeat what has been said. There is a growing conservatism in the treatment of diseases of the nasal passages, which argues well, not only for the patients, but for the profession; for part of our skill depends upon knowing when not to interfere with nature's own plan and method.

There is one suggestion in regard to the causation of the deformities of the septum, to which I invite your attention. Dr. Bosworth, of New York, is inclined to believe that they are all both deflections and deformities, due to traumatism. The injury may be received in infancy or childhood, and cause a chronic arthritis or deforming arthritis, which only shows itself to a degree demanding treatment later in life. Now, is it not possible that the formation of the interior of the nose, like the formation of its exterior, may be a family characteristic—in other words, due to heredity? The nose is said to be the feature that the laws of heredity most effect, and I see no reason why the interior, as well as the exterior, should not be under their influence. Traumatism is, no doubt, an important causative factor in the production of these deformities, but to say that they are all due to it seems to me to be a narrow position, in view of the possibility of the operation of other influences.

This suggestion as to the part heredity may play in the causation of these conditions, should make us cautious about interfering too much with the nasal passages, and tends to support the conservatism in regard to operations expressed in this paper.

A second rule to be borne in mind is, that there should always be sufficient breathing space through the nasal cavities. The nose is primarily an organ of respiration, and any interference with this function entails disastrous consequences. These evil results range from partial deafness to severe laryngeal and pulmonary disease. It is, therefore, most important that there should be no infringement upon the nasal breathing space.

A third rule has to do with drainage. Whenever there is sufficient obstruction to interfere with the removal of the nasal secretions, these become dry, through evaporation of their watery elements, and adhere tenaciously to the sides of the passages, causing considerable irritation. After a time, these crusts decompose, increasing the irritation and causing foul odors. It is, therefore, important to see that the proper drainage of the nose is not prevented in any way.

Though it sometimes happens that but one of these rules applies to a given case, they are generally found associated—a particular obstruction not only encroaching upon the breathing space, but causing considerable irritation, and interfering with proper drainage.

There are, of course, some special rules not coming within the province of this paper which bear upon this question, but the application of those we have given will generally enable you to determine whether or not the nasal passages are in need of treatment.

The conclusions of this paper can be formulated as follows:

The nasal tract requires treatment whenever there is within it (1) any cause of irritation; (2) any encroachment upon the normal breathing space; (3) any interference with its proper drainage.

